# Hsp90表达上调与肺癌细胞化疗耐药的相关性研究

**DOI:** 10.3779/j.issn.1009-3419.2011.06.02

**Published:** 2011-06-20

**Authors:** 婷 刘, 晓琨 王, 黎川 张

**Affiliations:** 116001 大连，大连大学附属中山医院呼吸内科 Department of Respiratory Medicine, the Afliated Zhongshan Hospital of Dalian University, Dalian 116001, China

**Keywords:** 肺肿瘤, Hsp90, GGA, 化疗耐药, Lung neoplasms, Hsp90, GGA, Drug resistance

## Abstract

**背景与目的:**

热休克蛋白（heat shock protein, Hsp）90是Hsps家族中的重要成员，其高表达与肿瘤的发生、发展及化疗耐药密切相关。本研究旨在探讨替普瑞酮（geranylgeranylacetone, GGA）作用下人肺癌细胞SPCA-1及H446中Hsp90的表达水平变化及与肺癌细胞对顺铂化疗耐药的相关性。

**方法:**

将细胞分成实验组与对照组，分别用含有不同浓度（0 μM, 10 μM, 50 μM, 100 μM, 500 μM, 1, 000 μM）的诱导剂GGA处理6 h。采用免疫荧光细胞化学及Western blot方法检测各组细胞中Hsp90在蛋白水平的表达；应用MTT法测定细胞在化疗药物顺铂作用下生存率，并分析Hsp90的表达在两种肺癌细胞对顺铂耐药中的作用。

**结果:**

SPCA-1及H446实验组细胞中Hsp90的表达水平均明显高于相应的对照组细胞，且与GGA具有一定的浓度依赖性。MTT显示两种实验组细胞对顺铂的生存率均明显高于相应的对照组细胞，亦呈一定的GGA浓度依赖性。

**结论:**

GGA可以诱导人肺癌细胞SPCA-1及H446中的Hsp90的表达上调，且其表达水平都与GGA具有一定的浓度依赖性。Hsp90高表达的细胞对顺铂的生存率明显高于低表达的细胞，表明Hsp90的表达上调与肺癌细胞对顺铂的耐药有一定的相关性。

## Abstract (en)

**Background and objective:**

Hsp90 is a major molecular chaperone, which overexpression is involved in oncogenesis, development and drug resistance in many human cancers. The aim of this study is to investigate the relationship between the GGA-induced overexpression of Hsp90 and chemoresistance to Cisplatin in SPCA-1 and H446 cell line.

**Methods:**

The protein expressions of Hsp90 induced by GGA at different concentrations were analyzed by Immunofluorescence and Western blotting. Cells survival to Cisplatin was determined using the MTT assays. The effect of Hsp90 expression on the drug resistance to Cisplatin in two Cell Lines was analyzed.

**Results:**

Compared with the respective control cells, Hsp90 expressions in both experimental cell lines were up-regulated obviously, exhibiting a dose-dependent manner to GGA. MTT assays revealed that the IC_50_s of cisplatin also showed a substantial elevation for the experimental cells of SPCA-1 and H446, and this elevation was also associated with GGA concerntration.

**Conclusion:**

GGA is effective for the induction of Hsp90 in the SPCA-1 and H446 cell line. Up-regulated Hsp90 is associated with the chemoresistance to Cisplatin in SPCA-1and H446 cells.

近年来，我国肺癌发病率逐年上升，分别占男性和女性恶性肿瘤的第一位和第二位，而化疗耐药是导致其治疗失败的主要原因之一。化疗的耐药性是多因素参与的过程，除了与肿瘤自身的基因类型有关外，还与肿瘤细胞所处的微环境密切相关^[1]^。肿瘤细胞在受到药物等不利因素刺激时，可诱导产生自身保护作用的热休克蛋白（heat shock protein, Hsp）。Hsps与肿瘤耐药性关系的研究已引起学者广泛的关注。Hsps多以分子伴侣的形式参与细胞的损失与修复，其高表达可增强肿瘤细胞的抗凋亡能力，并能引起细胞对化疗的耐药性增加^[2]^。Hsp90作为Hsps家族中拮抗细胞凋亡的主要蛋白，其众多底物蛋白参与了肿瘤细胞的生长调控及存活^[3]^，在促进肿瘤细胞增殖、抑制凋亡的信号转导过程中发挥重要作用。研究^[4-6]^表明Hsp90在肺癌、卵巢癌和乳腺癌等肿瘤细胞中呈高表达状态，并且其持续高表达的肿瘤细胞能对紫杉醇、顺铂和Doxorubicin等药物产生抗药性^[7]^，降低化疗的敏感性^[8]^。同样在白血病细胞中发现，高表达的Hsp90也参与肿瘤的耐药产生^[9]^。因此Hsp90已成为抗瘤治疗的研究热点。

萜类物质替普瑞酮（geranylgeranylacetone, GGA）作为Hsp70的一种选择性诱导剂，同样可以诱导调节Hsps中其它成员的表达。有研究^[10]^报道GGA能够诱导胃粘膜细胞的Hsp90表达上调。顺铂是肺癌治疗的一线药物，顺铂的耐药是临床治疗中不可忽视的问题。目前关于Hsp90的表达与肺癌细胞对顺铂化疗耐药性的研究报道较少。因此，本研究旨在通过应用GGA诱导两种肺癌细胞系中的Hsp90表达，并分析其在两种细胞系中表达的不同及与肺癌细胞对顺铂耐药的相关性。

## 1 材料与方法

### 1.1 材料与细胞

人肺腺癌标准细胞株SPCA-1及小细胞肺癌细胞株H446（均购自上海生科院细胞研究所的美国ATCC细胞），GGA、顺铂（Sigma公司），蛋白定量试剂盒、鼠抗人β-actin抗体（碧云天），FITC标记的羊抗小鼠抗体、HRP-羊抗兔、HRP-羊抗鼠IgG（北京中杉金桥），多克隆鼠抗、兔抗Hsp90抗体（美国Bioworld公司），RPMI-1640培养基（北京赛默飞化工）。

### 1.2 细胞培养及分组

5%CO_2_、饱和湿度、37 ℃恒温孵箱，于含10%胎牛血清、青霉素100 U/mL的RPMI-1640培养基培养SPCA-1及H446细胞，0.25%胰蛋白酶消化传代。取指数生长期的两种细胞各以5×10^4^个/mL细胞分别接种于100 mL的培养瓶中，长至80%融合时，弃去培养基，PBS液洗2次，待用。细胞随机分为实验组与对照组，参照文献^[11]^，实验组加入不同浓度GGA（10 μM、50 μM、100 μM、500 μM、1, 000 μM）；对照组加入PBS，培养6 h。然后分别采用免疫细胞荧光化学和Western blot检测两种细胞系各组细胞中Hsp90蛋白水平的表达。

### 1.3 免疫荧光细胞化学检测Hsp90蛋白水平的表达

消化收集对数生长期两种细胞系细胞，于六孔板中的盖玻片上各以1×10^5^/mL进行细胞爬片，细胞贴壁固定后，加适量培养基再培养24 h后随机按实验组和对照组分别处理分组，PBS冲洗，用4%多聚甲醛固定各组细胞15 min，用PBS冲洗3次后，0.2%Triton透膜15 min、0.1%PBST冲洗2次，再用1%BSA封闭30 min，分别加入一抗（多克隆鼠抗人Hsp90抗体1:200），4 ℃湿盒内过夜。PBS冲洗后，避光加入二抗（FITC标记的羊抗鼠抗体1:100），37 ℃杂交1 h。最后PBS冲洗，滤纸吸干后封片。在激光共聚焦488 nm处观察各组细胞Hsp90出现的位置及强度变化。

### 1.4 Western blot法检测Hsp90蛋白水平的表达

消化收集两种细胞系的各培养瓶中细胞于1.5 mL Ep管中，加入含10 μL/mL的蛋白酶抑制剂PMSF的RIPA裂解液400 μL，置冰上裂解30 min，以12, 000 rpm、4 ℃离心15 min，取上清转移至新管，重复离心2次，-70 ℃保存待测；BCA蛋白定量试剂盒测定蛋白含量，进行SDS-PAGE恒压100 V电泳90 min、恒流300 mA湿式电转印2 h。将转印的PVDF膜放入封闭液中4 ℃过夜，分别加入稀释的兔抗人Hsp90抗体（一抗，1:500）及鼠抗人β-actin（一抗，1:400），37 ℃孵育2 h，PBST洗膜3次，每次5 min。再加入稀释的辣根过氧化物酶标记羊抗兔、羊抗鼠IgG（二抗，1:2, 000），37 ℃孵育1 h，PBST洗膜3次，每次5 min，室温化学发光显色。凝胶成像系统对PVDF膜进行扫描分析，计算公式为：某样品Hsp90的相对表达水平=样品Hsp90的IOD值/样品β-actin的IOD值。

### 1.5 MTT法检测GGA诱导对细胞生存率的影响

取对数生长期两种细胞系的各组细胞，分别以1×10^4^个/孔的密度接种于96孔培养板，每孔加200 μL培养基，置于5%CO_2_、饱和湿度的37 ℃孵箱培养24 h。加入不同浓度的顺铂（0 μM、2 μM、4 μM、6 μM、8 μM），每个浓度设3个孔，继续培养顺铂组48 h后，再向各孔加入MT 20 μL，4 h后加入DMSO，酶标仪上选择波长490 nm，检测各孔OD值，计算细胞生存率。细胞生存率=（实验组OD值-空白对照组OD值）/（对照组OD值-空白对照组OD值）× 100%。应用Modifed Kaber method法求出细胞的半数抑制浓度（50% inhibitory concentriton, IC_50_），并求得耐药指数。耐药指数（resistance index, RI）=IC_50_实验组/IC_50_对照组。

### 1.6 统计学处理

采用SPSS 11.5统计软件，所有实验均重复3次以上，实验数据用Mean±SD表示，采用单因素方差分析比较组间差异，*P* < 0.05为差异有统计学意义。

## 2 结果

### 2.1 Hsp90蛋白水平的表达

采用免疫荧光化学的方法，比较实验组与对照组细胞Hsp90蛋白的表达。用FITC染色后，在488 nm激发光的激发下，NIKON C1激光共聚焦显微镜下观察，在两种细胞系中均可见该蛋白主要分布于细胞核周围及胞浆中。与相对应的对照组相比，两种细胞系的各实验组细胞的荧光强度均随GGA浓度的增加呈现明显增强趋势，Hsp90蛋白表达逐渐增加（图 1）。

**1:**
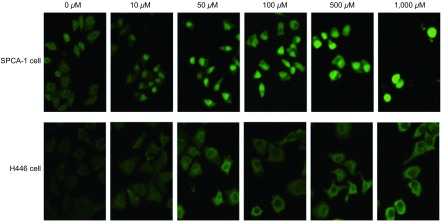
免疫荧光细胞化学法检测SPCA-1及H446细胞中不同浓度GGA作用下的Hsp90蛋白表达（×400） Expression of Hsp90 protein detected by immunofluorescence in SPCA-1 and H446 cells (×400)

为了进一步确定Hsp90的蛋白表达水平变化的差异，采用Western blot杂交的方法来检测SPCA-1及H446各组细胞中Hsp90的相对表达量（图 2，图 3）。表 1显示，SPCA-1细胞中，当GGA的浓度为10 μM-1, 000 μM时，Hsp90蛋白表达量逐渐增高且随GGA呈浓度依赖性增高（除了10 μM与0 μM比较无统计学差别外），其表达水平与对照组相比提高了0.2倍-1.0倍。与SPCA-1细胞相似，在H446细胞中Hsp90蛋白表达量也与GGA呈浓度依赖性增加，分别与对照组相比提高了0.6倍-2.1倍（GGA浓度为100 μM-1, 000 μM时）。

**2:**
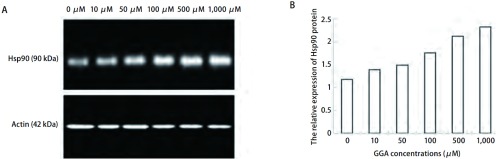
不同浓度GGA诱导下SPCA-1细胞中Hsp90蛋白水平的表达。A：Western blot测定不同GGA浓度（0 *μ*M, 10 *μ*M, 50 *μ*M, 100 *μ*M, 500 *μ*M, 1, 000 *μ*M）作用6 h后SPCA-1细胞中的Hsp90蛋白水平的表达；B：柱状图示Hsp90相对*β*-actin的表达量。 Expression of Hsp90 protein in SPCA-1 cells exposed to GGA at different concentrations. A: Western blot results of Hsp90 protein in SPCA-1 cells exposed to GGA at different concentrations (0 *μ*M, 10 *μ*M, 50 *μ*M, 100 *μ*M, 500 *μ*M, 1, 000 *μ*M) for 6 h; B: Bar graph showing the relative level of Hsp90 protein evaluated using the ratio of of IOD_Hsp90_/IOD_*β*-actin_.

**3:**
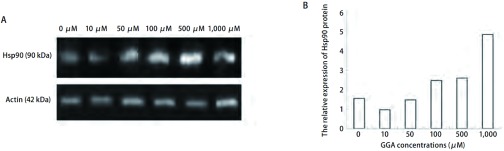
不同浓度GGA诱导下H446细胞中Hsp90蛋白水平的表达。A：Western blot测定不同GGA浓度（0 *μ*M, 10 *μ*M, 50 *μ*M, 100 *μ*M, 500 *μ*M, 1, 000 *μ*M）作用6 h后H446细胞中的Hsp90蛋白水平的表达；B：柱状图示Hsp90相对*β*-actin的表达量。 Expression of Hsp90 protein in H446 cells exposed to GGA at different concentrations. A: Western blot results of Hsp90 protein in H446 cells exposed to GGA at different concentrations (0 *μ*M, 10 *μ*M, 50 *μ*M, 100 *μ*M, 500 *μ*M, 1, 000 *μ*M) for 6 h; B: Bar graph showing the relative level of Hsp90 protein evaluated using the ratio of of IOD_Hsp90_/IOD_*β*-actin_.

**1:** 不同浓度GGA诱导下SPCA-1、H446细胞Hsp90蛋白水平的相对表达 Expression of Hsp90 protein in SPCA-1 and H446 cells exposed to GGA at different concentrations

GGA (*μ*M)	OD	
	SPCA-1	H446
0	1.180,0±0.113,6^*^	1.558,9±0.148,0^*^
10	1.393,8±0.178,9^#^	0.974,9±0.052,0^*^
50	1.494,6±0.113,6^*^	1.482,6±0.087,8^*^
100	1.757,7±0.227,9^*^	2.477,8±0.123,9^*^
500	2.127,7±0.161,4^*^	2.601,5±0.135,0^*^
1,000	2.331,3±0.098,8^*^	4.847,8±0.262,3^*^
Graph showing the relative level of Hsp90 protein evaluated using the ratio of IOD_Hsp90_/IOD_*β*-actin_. Compare with 0 *μ*M,^*^P < 0.05,^#^*P* > 0.05.		

GGA可以诱导人肺腺癌细胞SPCA-1及小细胞肺癌细胞H446中Hsp90的表达。两种细胞系中，在一定范围内，Hsp90的表达在蛋白水平随诱导剂呈浓度依赖性的上调。在SPCA-1细胞，当GGA浓度在50 μM时开始出现诱导效应，在1, 000 μM时达到最大效应（与对照组相比提高了1.0倍）；在H446细胞中，Hsp90的表达与其相似，但在GGA浓度为100 μM时才开始出现诱导效应，在1, 000 μM时达到最大效应（与对照组相比提高了2.1倍）。统计学分析显示，在非小细胞肺癌SPCA-1中，Hsp90的表达与诱导剂的浓度梯度依赖性更明显。

### 2.2 GGA诱导对细胞生存率的影响

为了研究GGA诱导Hsp90的表达与两种细胞对顺铂敏感性的相关性比较了不同浓度GGA（0 μM-1, 000 μM）诱导下细胞的IC_50_值。结合细胞在不同浓度顺铂（0 μM、2 μM、4 μM、6 μM、8 μM）作用下的生存曲线（图 4），在SPCA-1细胞中，与对照组IC_50_值4.74±0.23相比，当GGA浓度依次为10 μM、50 μM、100 μM、500 μM、1, 000 μM时，实验组IC_50_的值分别为：5.71±0.11、6.27±0.77、6.42±0.03、7.12±0.02和7.25± 0.04。同样，在H446细胞中对照组的IC_50_值为4.28±0.22；GGA浓度依次增加时，实验组的IC_50_值分别为4.73±0.08、5.57±0.15、6.23±0.17、7.12±0.02和7.69±0.08。结果表明：在相同浓度顺铂作用下，两种肺癌细胞系GGA各诱导组细胞的耐药率均明显高于所对应的对照组（*P* < 0.05），且细胞的耐药性与Hsp90的表达量成正比。最大RI分别为1.52（SPCA-1细胞）及1.79（H446细胞）（GGA浓度为1, 000 μM）。在SPCA-1及H446两种细胞中，Hsp90的表达均与细胞对顺铂的耐药性有关。

**4:**
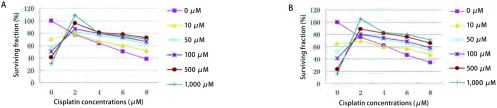
不同浓度GGA诱导下的SPCA-1及H446细胞对顺铂的生存曲线。A：图示不同GGA浓度（0 *μ*M, 10 *μ*M, 50 *μ*M, 100 *μ*M, 500 *μ*M, 1, 000 *μ*M）作用6 h后SPCA-1细胞对顺铂的生存率；B：图示不同GGA浓度（0 *μ*M, 10 *μ*M, 50 *μ*M, 100 *μ*M, 500 *μ*M, 1, 000 *μ*M）作用6 h后H446细胞对顺铂的生存率。 Survival curves of SPCA-1 and H446 cells to cisplatin after being induced by GGA at different concentrations. A: Graph showing the survival curves of SPCA-1 cells to cisplatin after being induced by GGA at different concentrations (0 *μ*M, 10 *μ*M, 50 *μ*M, 100 *μ*M, 500 *μ*M, 1, 000 *μ*M) for 6 h; B: Graph showing the survival curves of H446 cells to cisplatin after being induced by GGA at different concentrations (0 *μ*M, 10 *μ*M, 50 *μ*M, 100 *μ*M, 500 *μ*M, 1, 000 *μ*M) for 6 h.

## 3 讨论

化疗耐药是肺癌现阶段治疗中难以取得实质性进展的主要障碍。肺癌的耐药原因涉及多种机制，主要分为原发性耐药和继发性耐药，前者表现为肿瘤细胞对化疗药物的天然不敏感，后者是肿瘤细胞因抗癌药物诱导或其他因素的激活而产生的耐药性。Hsps作为一种具有重要生理功能的应激蛋白，已经成为肿瘤化疗耐药的研究热点之一。Hsp90是家族重要成员，以“分子伴侣”的形式存在于细胞内，温度、蛋白错误折叠、感染和癌等各种应激因子可影响其合成^[12]^。Hsp90在应激条件下的高表达可维持细胞必需的蛋白质空间构象，保护细胞的功能、生存和对应激原的耐受，是细胞的一种保护机制。研究^[13-15]^发现，Hsp90与凋亡关系密切。Hsp90可以通过多种机制抑制细胞凋亡的发生。此外，很多研究通过抑制^[11, 16]^及反义RNA^[7]^等多种方法均证实HSP90对细胞具有抗凋亡作用。

本研究通过GG A诱导肺癌SPCA-1及H446细胞中Hsp90的表达：当GGA浓度为10 μM-1, 000 μM时，两种细胞系中Hsp90蛋白水平的表达明显升高，并与诱导剂的浓度呈现一定依赖性。GGA作为一种非毒性的诱导剂，无论是体内还是在体外都能诱导Hsp70的表达^[17-19]^。国外的研究^[20]^发现，在神经细胞中，GGA同样也可以诱导Hsp90的高表达，其机理可能是通过促使Hsp90与热休克因子（human heat shock factor 1, HSF）的解离，导致多种HSF释放并进入细胞核，使HSF1在胞浆内以非DNA结合活性的单体形式游离出来，发生磷酸化后进入胞核，促进*Hsp*基因转录和翻译过程，从而诱导Hsp90快速、过量表达。

研究^[21-25]^认为Hsp90在肿瘤细胞的存活过程中有非常重要的作用，已成为抗肿瘤治疗的新靶点。但是，目前关于Hsp90与肺癌化疗耐药的研究还不多见，我们选取了临床常用化疗药物顺铂及高发病率的人肺腺癌细胞作为本次研究对象。顺铂是周期非特异性药物，它主要通过与DNA分子形成链内或链间交叉联接或阻止RNA分子再复制等途径发挥抗肿瘤作用。结合国外已有的研究成果^[11]^，本研究主要应用不同浓度的GGA诱导后人肺癌细胞中Hsp90的表达，并采用有较高的临床参考价值的MT比色法^[26-28]^，分析Hsp90高表达在顺铂耐药中的作用。结果表明：与对照组相比，两种细胞系中，Hsp90高表达组（GGA诱导组）的细胞对顺铂的耐药率增加，且与Hsp90表达水平呈正相关。即Hsp90高表达有助于肺癌细胞对化疗药物顺铂产生耐药性。Hsp90上调引起肿瘤细胞耐药性增加可能与如下的原因有关：Hsp90通过其受体多环节多途径影响癌细胞生长和（或）存活。此外，Hsp90利用其与凋亡的密切关系^[29]^，启动下游多种作用蛋白，参与调节不同的信号转导通路，抑制凋亡的启动，从而使细胞对化疗药物诱导的凋亡产生抗性^[30]^。该实验结果对于指导临床化疗具有一定参考价值。

当然，肺癌细胞对化疗药物的耐药是由多因素多机制共同作用的结果，细胞中Hsp90参与作用外，还有细胞自身对化疗药物的天然抗药性，以及多药耐药蛋白等机制共同作用。因此，进一步研究Hsp90在肺癌耐药中的表达规律及与预后关系，可能为临床合理的治疗提供一定的理论依据。
